# Wasp venom-induced acute kidney injury: current progress and prospects

**DOI:** 10.1080/0886022X.2023.2259230

**Published:** 2023-09-19

**Authors:** Fanglin Yu, Ling Wang, Hai Yuan, Zhao Gao, Li He, Fengqi Hu

**Affiliations:** a School of Medicine, Wuhan University of Science and Technology, Wuhan, China; b Department of Nephrology, Xiangyang Central Hospital, Affiliated Hospital of Hubei University of Arts and Science, Xiangyang, China

**Keywords:** Acute kidney injury, cilastatin, therapeutic plasma exchange, varespladib, wasp venom, wasp sting

## Abstract

Wasp venom can trigger local and systemic reactions, with the kidneys being commonly affected, potentially causing acute kidney injury (AKI). Despite of the recent advances, our knowledge on the underlying mechanisms of toxicity and targeted therapies remain poor. AKI can result from direct nephrotoxic effects of the wasp venom or secondary rhabdomyolysis and intravascular hemolysis, which will release myoglobin and free hemoglobin. Inflammatory responses play a central role in these pathological mechanisms. Noteworthily, the successful establishment of a suitable experimental model can assist in basic research and clinical advancements related to wasp venom-induced AKI. The combination of therapeutic plasma exchange and continuous renal replacement therapy appears to be the preferred treatment for wasp venom-induced AKI. In addition, studies on cilastatin and varespladib for wasp venom-induced AKI treatment have shown their potential as therapeutic agents. This review summarizes the available evidence on the mechanisms and treatment of wasp venom-induced AKI, with a particular focus on the role of inflammatory responses and potential targets for therapeutic drugs, and, therefore, aiming to support the development of clinical treatment against wasp venom-induced AKI.

## Graphical Abstract

**Figure:**
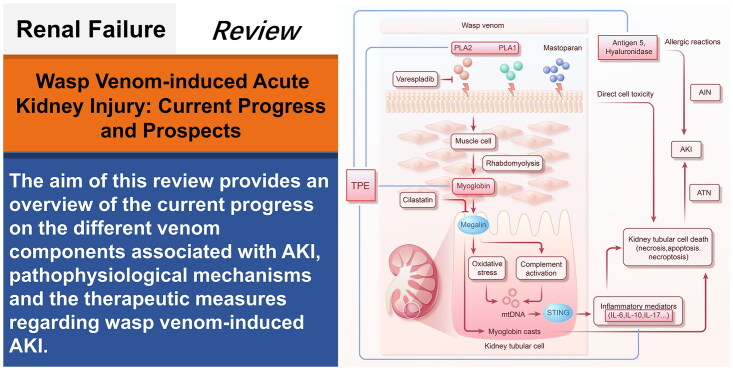

## 1. Introduction

Wasps belong to the order Hymenoptera [1].Wasp stings mainly occur in mountain forests far from cities; however, they are becoming increasingly common (in urban and suburban wilderness interfaces) worldwide and are a growing public health problem [2–4]. The kidney is one of the most commonly affected organs upon a wasp sting [1]. Studies have shown that the incidence of acute kidney injury (AKI) is as high as 30–50% in patients with wasp stings [5]. The content and respective amount in the venom vary among different wasp species; nonetheless, their main components are similar [6,7]. Currently, no systematic comparison of the venom of different wasp species concerning their specific toxicological profiles is available. The composition of wasp venom, which includes proteins, enzymes, bioactive peptides, and amines, has been well investigated [1,8]. Compared with those having AKI following snakebite and bee stings, deaths are more common among wasp sting victims with AKI [9]. Another study indicated that wasp stings generally inflict more severe outcomes than bee stings [10]. The progression of wasp venom-induced AKI is rapid and delayed admission is an independent risk factor for AKI following a wasp sting [11,12], which can seriously impact clinical progress. In a retrospective cohort study of 112 patients with wasp stings, no patients without AKI died [13]. Clinical studies have also shown that AKI in patients with wasp stings is an independent risk factor for poor prognosis [13,14]. Wasp venom-induced AKI is relatively easy to diagnose if there is a history of a wasp sting and the AKI criteria are met, such as the Kidney Disease Improving Global Outcomes criteria. However, the underlying mechanism of wasp venom-induced AKI is not fully understood. Therefore, there are no specific treatments targeting the pathogenic processes induced by wasp venom, with treatment being mainly supportive and mortality being as high as 5.6–50% [3].

**Figure 1.**
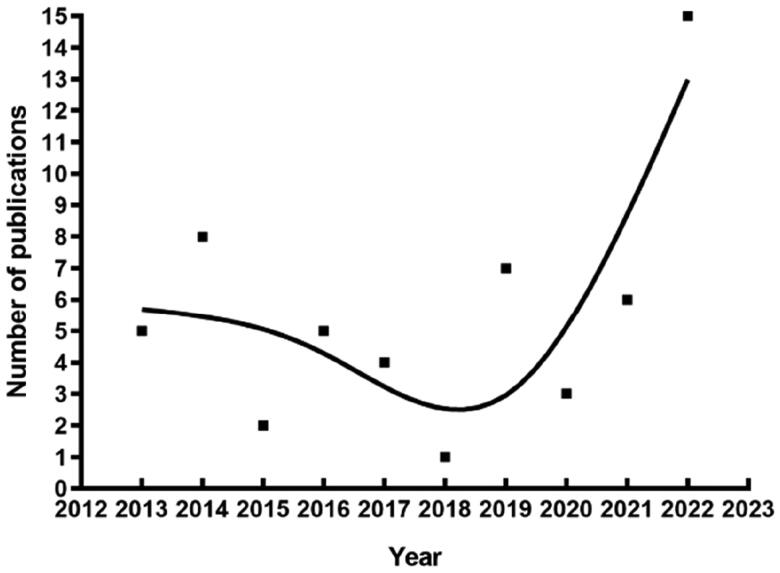
Number of publications per year in recent decades retrieved from PubMed (https://pubmed.ncbi.nlm.nih.gov/) using ‘wasp and (acute kidney injury or acute renal failure)’ as keywords.

Wasp venom-induced AKI has attracted increasing attention in recent years, with studies related to wasp venom-induced AKI rapidly increasing [11], especially in the past 10 years (Figure 1).

In 2022 alone, 15 articles related to wasp venom-induced AKI were published, which is the highest number in a single year in the PubMed database records. Recent advances in wasp venom-induced AKI have focused on the mechanisms, application of therapeutic plasma exchange (TPE), and potential therapeutic drugs. Therefore, the aim of this review was to provide an overview of the current progress on the different venom components potentially related with AKI development, as well as on the pathophysiological mechanisms of and therapeutic approaches for wasp venom-induced AKI.

## 2. Wasp venom components associated with AKI (Table 1)

### 2.1. Phospholipase A (PLA)

PLA is one of the main wasp venom components and it can hydrolyze phospholipid molecules on cell membranes; thus, it can disrupt the integrity of cell membranes and induce cell rupture [15,16]. PLA acts on cell membranes of muscle cells, red blood cells, and renal tubular epithelial cells [17,18]. Its action on muscle cells and red blood cells causes rhabdomyolysis and intravascular hemolysis, which results in the release of myoglobin and free hemoglobin [17,19]. In addition, PLA acts directly on renal tubular epithelial cells by damaging cell membrane integrity, leading to direct renal cytotoxicity [17,18]. According to different substrate cleavage sites, PLA can be classified into subtypes, including PLA1 and PLA2 that hydrolyze the sn-1 and sn-2 esters of glycerophospholipids, respectively [20]. Furthermore, PLA is regarded as an allergen in IgE-mediated allergic reactions [15,21].

**Table 1.** Description of the components of wasp venom and their pathophysiological effects contributing for AKI development.

Component name	Function	AKI pathophysiology
PLA	Disrupt the cell membrane integrity	Rhabdomyolysis, intravascular hemolysis, direct renal toxicity, allergic reactions, and inflammatory response
Mastoparan	Instability of the cell membrane structure	Rhabdomyolysis, intravascular hemolysis, and direct renal toxicity
Antigen 5	Allergen	Allergic reactions
Hyaluronidase	Allergen and spreading factor	Allergic reactions

AKI, acute kidney injury; PLA, phospholipase A.

### 2.2. Mastoparan

Mastoparan is a 14-amino acid amphiphilic peptide with both hydrophobic and hydrophilic residues that form amphipathic helical structures [22]. Hydrophobic residues can be embedded into the lipid bilayer of the cell membrane, and hydrophilic residues can form pores on the cell membrane, causing instability of the cell membrane structure and increasing its permeability, thereby leading to cell lysis and inactivation [6,17,23]. Mastoparan can activate G proteins on the cell membrane surface, thus disrupting transmembrane signals, activating phospholipase, mobilizing Ca^2+^ from mitochondria and the sarcoplasmic reticulum, activating various regulatory enzymes, such as Na^+^-K^+^-ATPase, causing changes in mitochondrial permeability, and participating in cell necrosis or apoptosis [15,20,24]. Mastoparan has various biological activities; for examples, it can activate guanylate cyclase in addition to G proteins and phospholipases, leading to muscle cell and renal tubular cell lysis [6,25]. In pharmacology, mastoparan is also important owing its antibacterial, antiviral, and antitumor effects [20,22]; however, its application is limited due to its strong hemolytic toxicity and cytotoxicity [26].

### 2.3. Antigen 5

Antigen 5 present in wasp venom is an important cause of allergic reactions [21]. It exists only in the wasp family and not in the bee family. Therefore, its presence can be used to distinguish wasps from bees [27].

### 2.4. Hyaluronidase

Hyaluronidase is one of the major allergens in wasp venom [3,6]. Hyaluronidase is considered the most conserved allergen in Hymenoptera and the important cross-reactive allergen in wasp venom [28]. Hyaluronidase, also known as the ‘spreading factor’, is a major component of hydrolyzed extracellular matrix. The viscous polymer hyaluronic acid is hydrolyzed by hyaluronidase into non-viscous fragments, contributing to pro-inflammatory, pro-angiogenic, and immunostimulating effects [15,17,29]. In addition, hyaluronidase-induced hydrolysis of hyaluronic acid in the extracellular matrix allows the venom to penetrate different tissues and thus facilitates the diffusion of the venom into circulation, thereby enhancing the effects of other venom components [17].

## 3. From clinical renal biopsy to an animal model

### 3.1. Implications of clinical renal biopsy

Tissue biopsy remains the gold standard for the assessment of renal deterioration, including AKI. However, this diagnostic approach is limited due to the commonly severe preoperative condition of the patients (due to abnormal coagulation function) and uneven distribution of medical resources in remote areas. Therefore, fewer than 30 patients with wasp venom-induced AKI who underwent renal biopsy have been reported in the literature (Table 2).

**Table 2.** Summary of studies on wasp venom-induced AKI.^a^

Study	Rhabdomyolysis	Hemolysis	No. of renal biopsy cases	Outcome (*n*)
Sitprijafrs and Boonpucknavig [30]	Yes	No	1	ATN (1)
Vachvanichsanong et al. [31]	No	Yes	1	AIN (1)
Thiruventhiran et al. [32]	No	Yes	1	ATN (1)
Zhang et al. [33]	Yes	Yes	1	AIN (1)
Chao et al. [34]	Yes	Yes	1	ATN + AIN (1)
Nandi and Sarkar [35]	No	No	1	AIN (1)
Natarajan et al. [36]	Yes	Yes	1	ATN (1)
Dhanapriya et al. [37]	Yes	Yes	4	ATN (2), AIN (1), ATN + AIN (1)
Vikrant and Parashar [38]	Yes	Yes	13	ATN (4), AIN (3), ATN + AIN (6)
Ambarsari et al. [39]	No	Yes	2	ATN + AIN (2)
Ou et al. [40]	Yes	Yes	1	ATN (1)

AIN, acute interstitial nephritis; AKI, acute kidney injury; ATN, acute tubular necrosis.

^a^Data obtained based on histological patterns observed among patients who underwent renal biopsy.

Most of these articles were case reports and lacked a systematic analysis of the pathological changes in the kidney. The largest sample size study enrolled 13 patients with wasp venom-induced AKI who underwent renal biopsy [38] and reported that acute tubular necrosis (ATN) with or without pigmented granular cast was the most common lesion (77%) and that acute interstitial nephritis (AIN) alone occurred in 23% of patients. ATN in patients with wasp venom-induced AKI is characterized by tubular dilatation, brush border loss, epithelial detachment, and cast formation [39,40]. Further studies confirmed, through immunohistochemistry, that the pigmented granular cast in the renal tubule was composed of myoglobin, hemoglobin, or both [34,36]. However, until recently, the signaling pathway linking ATN to wasp venom was poorly defined. Recent advances, including the development of an animal model related to wasp venom-induced AKI and other *in vitro* approaches, have begun to unveil the mechanisms of wasp venom-induced ATN.

### 3.2. Risk factors related of wasp venom-induced AKI

Assessments of risk factors aid the early detection, diagnosis, and early treatment of any health disorder, including wasp venom-induced AKI. Indeed, previous studies showed that rhabdomyolysis indicators (elevated myoglobin and aspartate aminotransferase), hemolysis indicators (levels of lactate dehydrogenase, LDH), inflammation indicators (number of leukocytes, elevated urinary monocytes chemical protein [MCP]-1), number of stings, time from stings to admission, and activated partial thromboplastin time are independent risk factors for wasp venom-induced AKI [4,12,13].

### 3.3. Animal model related to wasp venom-induced AKI

In 2022, we established an animal model of AKI by subcutaneously injecting wasp venom (*Vespa mandarinia,* also known as Asian giant hornets). This model successfully reproduced the clinical manifestations associated with multiple wasp stings [5]. We observed that rats with wasp venom-induced AKI demonstrated increased serum creatinine and creatine kinase (CK) levels, decreased glomerular filtration rate, oliguria, myoglobinuria, and ATN [5]. The histopathological changes were typical of ATN with loss of tubular dilatation, tubule brush border, tubular epithelial cell sloughing, and cast formation [5,41]. AKI pathogenesis in the wasp venom-induced AKI *in vivo* model was confirmed to be secondary to rhabdomyolysis and intravascular hemolysis [5]. In addition, using this model, we further demonstrated that myoglobin reabsorption and PLA2 may be potential targets for treating wasp venom-induced AKI [41,42]. Tang et al. [43] also successfully established a PLA2-induced AKI model using PLA2 injected into mice *via* their tail vein to reproduce the clinical manifestations of wasp sting [43]. The successful establishment of a wasp venom-induced AKI model can support basic and clinical research onto wasp venom-induced AKI.

## 4. AKI pathophysiology induced by wasp venom

### 4.1. Secondary to rhabdomyolysis

In wasp sting clinical studies, elevated levels of myoglobin (in blood, urine, or both) and CK are typically used as biomarkers of rhabdomyolysis [14,16,44]. Several large-scale clinical studies suggested that rhabdomyolysis is more prevalent than intravascular hemolysis in patients who suffered wasp sting [4,14]. A previous study also found a higher incidence of AKI in patients with wasp stings accompanied by rhabdomyolysis [14]. Further regression analysis showed that rhabdomyolysis on admission was an independent risk factor for wasp venom-induced AKI [13]. Therefore, some scholars have suggested wasp venom-induced AKI to be a non-classical rhabdomyolysis-induced AKI [1,14,37]. We are inclined to agree with that interpretation and suppose that rhabdomyolysis was the primary mechanism of wasp venom-induced AKI. Our recent animal study further demonstrated that myoglobin is one of the most upregulated genes in the AKI group compared with the normal group [42].

Although the underlying mechanism between wasp venom and rhabdomyolysis remains unclear, recent related studies provide valuable clues and a basis for revealing the role of rhabdomyolysis in wasp venom. Proteomics and metabolomics data indicate that AKI secondary to rhabdomyolysis is highly associated to the disruption of phospholipid molecules on muscle cell membranes [16]. As described above, PLA within wasp venom can attack the muscle cell membrane, damaging muscle cells and releasing myoglobin into circulation, leading to rhabdomyolysis [17,19]. Additionally, mastoparan can disrupt the phosphorylation of proteins essential for muscle cell membrane stabilization, and interfere with mitochondrial and sarcoplasmic reticulum Ca^2+^ mobilization, thereby affecting the sarcoplasmic reticulum and mitochondrial structure and function, leading to rhabdomyolysis [24].

Rhabdomyolysis leads to myoglobin release. Several distinct mechanisms by which myoglobin causes renal toxicity include renal vasoconstriction, direct toxicity of myoglobin to renal tubule cells, and formation of intratubular casts [45]. When in circulation, myoglobin induces renal vasoconstriction by removing nitric oxide, consequently reduces intravascular blood volume and leads to renal hypoperfusion or ischemia, and ultimately promotes AKI [3,46]. Then, myoglobin circulates to the kidneys and filters freely from the glomerulus into the tubules [45], where it is reabsorbed by proximal renal tubule cells *via* endocytic receptors, such as megalin [47]. Noteworthily, proximal renal tubular cells are particularly susceptible to injury from nephrotoxic myoglobin [48–50]. Direct cytotoxic effects associated with myoglobin have been ascribed to oxidative injury [46]. Moreover, myoglobin binds to the Tam-Horsfall protein and forms a precipitate in the distal renal tubules [45], which forms pigmented granular casts that cause tubular obstruction [51], that will lead to increased pressure within the kidney tubules, exceeding the interstitial pressure. This results in reduced vascular inflow and perfusion, ultimately leading to AKI [52].

### 4.2. Secondary to intravascular hemolysis

Previous studies have also indicated a higher incidence of AKI in patients with hemolysis associated with wasp stings, suggesting a potential involvement of hemolysis in the development of wasp venom-induced AKI [14,16]. PLA and mastoparan can act on the red blood cell membrane, contributing to hemoglobin release that will result in intravascular hemolysis [17,19]. Free hemoglobin can cause adverse clinical effects associated with direct cytotoxicity, vasoconstriction, intratubular casts formation, inflammation, complement activation, and oxidative reactions (lipid peroxidation and mitochondrial dysfunction) [53,54]. Currently, research on intravascular hemolysis associated with wasp venom-induced AKI is far less than that on rhabdomyolysis. In addition, there is a lack of reliable commercial kits to measure free hemoglobin levels since the kidney is a blood-rich organ, and it is technically difficult to remove all non-megalin-binding hemoglobin while retaining megalin-binding hemoglobin. Hemoglobin immunohistochemistry in the renal tubules was unconfirmed in most cases [55]. These factors have hindered the study of intravascular hemolysis in the context of wasp venom-induced AKI.

### 4.3. Direct toxicity to the kidney

Since various venoms are reabsorbed in the renal tubules, this makes proximal epithelial cells particularly susceptible. As mentioned earlier, PLA and mastoparan in wasp venom can act on renal tubule epithelial cell membranes and cause direct damage. Tang et al. [43] suggested that PLA2 induces mitochondrial apoptosis in renal tubular epithelial cells, thus playing a role in the development of wasp venom-induced AKI [43].

### 4.4. Allergic reactions

Allergic reactions to wasp venom resulting in AIN have been reported [38]. AIN is another cause of wasp venom-induced AKI (Table 2). Allergenic components of wasp venom (PLA, hyaluronidase, and antigen 5) can trigger type I hypersensitivity reactions by IgE-mediated and type III hypersensitivity reactions [56,57]. This information provides a theoretical basis for the application of glucocorticoids in the treatment of wasp venom-induced AKI, especially when pathological evidence of AIN is confirmed by renal biopsy. Notably, serious systemic allergic reactions, such as anaphylactic shock and laryngeal edema, are not uncommon in wasp sting patients, for which glucocorticoids are recommended regardless of the presence of AKI (when there are no contraindications). However, in-depth studies on wasp venom are lacking, and the optimal doses and courses of glucocorticoids to use to treat wasp venom-induced AKI are to be defined.

### 4.5. Inflammatory response

Previous studies have shown that the inflammatory response is a common feature and plays an important role in AKI pathogenesis [58]. Studies have shown that patients with wasp stings develop systemic inflammation [59]. Li et al. [60] reported that the levels of inflammatory mediators (such as interleukin [IL]-6, IL-10, and IL-17) are increased in patients who developed wasp venom-induced multiple organ dysfunction syndrome and that the levels of some of these inflammatory mediators (IL-2, IL-4, IL-10, IL-17, and interferon [IFN]-γ) correlate positively with kidney dysfunction [60]. Previous study also indicated that AKI patients have high levels of serum leukocytes and urinary MCP-1, which are independent risk factors for AKI [13]. Transcriptomics data in wasp venom-induced animal AKI model have revealed that the expression of inflammatory genes in the kidney is increased and further pathway analyses showed that the inflammatory response pathway is highly enriched [42]. All these studies emphasize the role of inflammation in wasp venom-induced AKI.

Stimulator of interferon genes (STING) is a key regulator of inflammation in ischemia-reperfusion injury-induced and cisplatin-induced AKI [61–64]. However, it remains unclear whether the STING signaling pathway is activated and plays a role in the development of wasp venom-induced AKI. More recently, STING knockout or pharmacological inhibition *in vivo* data, as well as STING knockdown *in vitro* data, revealed that STING-TBK1/IRF3 signaling pathway plays a central role in regulating the inflammatory response and inflammation mediators (IL-6, MCP-1, IFN-α, and IFN-β) in wasp venom-induced AKI [65]. Further studies are needed to explore the mechanism of STING activation in wasp venom-induced AKI. Nonetheless, previous studies indicate that STING activation is associated with mitochondrial DNA (mtDNA) leakage from the kidney and its release in urine [42,66], a process that is also associated with myoglobin-induced mitochondrial lipid peroxidation, oxidative stress of mitochondria, and mitochondria apoptosis [49,67,68]. Additionally, myoglobin can impair the mitochondrial membrane potential and increase the levels of reactive oxygen species and lipid peroxidation in mitochondria of the renal tubular epithelial cells [49]. This mechanism is supported by the activation of markers of oxidative stress (malondialdehyde and superoxide dismutase), which was described in rats with wasp venom-induced AKI [41].

**Figure 2.**
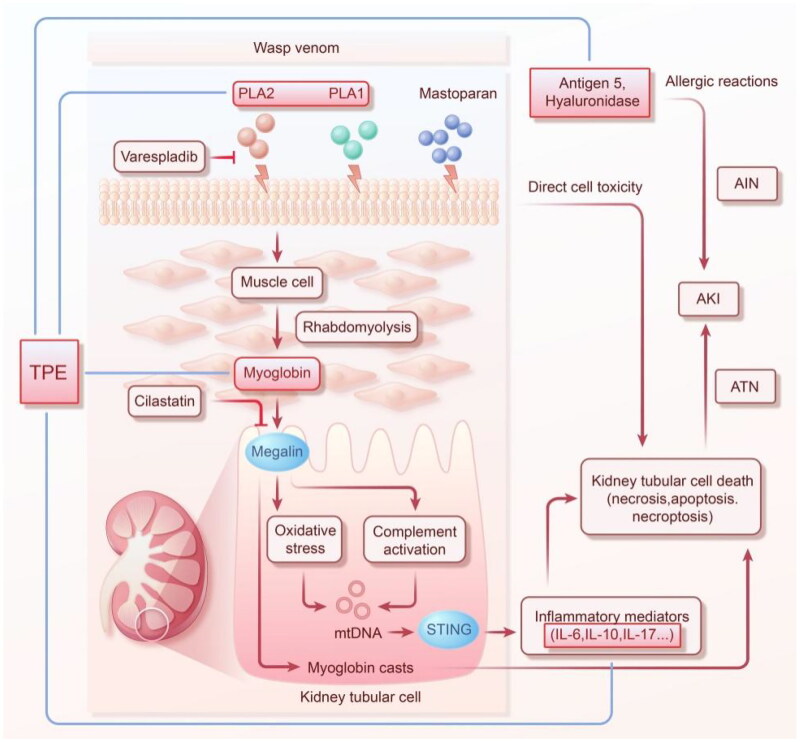
Schematic diagram of the mechanism and treatment of wasp venom-induced AKI. AIN, acute interstitial nephritis; AKI, acute kidney injury; ATN, acute tubular necrosis; IL, interleukin; mtDNA, mitochondrial DNA; PLA, phospholipase A; STING, stimulator of interferon genes; TPE, therapeutic plasma exchange.

In renal tubular epithelial cells, PLA2 can induce complement-mediated mitochondrial apoptosis [43]. Indeed, the complement system was shown to play a very important role in rhabdomyolysis- and intravascular hemolysis-induced AKI [69–72]. The activation of the complement system is mainly through the classical, lectin, and bypass pathways, among which C3 is the central molecule of the complement system and the common pathway of these three activation pathways [73]. Our recent study also described that complement C3 is deposited in the renal tubules of rats with wasp venom-induced AKI, further indicating that wasp venom-induced AKI activates the complement system [41]. However, the specific pathway of complement activation in wasp sting-induced AKI remains unclear (Figure 2).

## 5. Therapeutic measures

### 5.1. Therapeutic plasma exchange (TPE)

Wasp stings induce AKI in patients who are typically admitted to the nephrology department, where doctors and nurses are naturally familiar with TPE. Unlike dialysis or hemofiltration, which removes pathogenic molecules of low to medium molecular weight (normally <10 kDa, such as water, creatinine, urea, and potassium) from the serum, TPE is a blood purification technique that removes large molecular weight pathogenic molecules (>15 kDa) present in the plasma, such as immune complexes, pathogenic proteins, and inflammation mediators in circulating blood [74,75]. TPE is an important treatment approach for various kidney diseases, such as anti-neutrophil cytoplasmic antibody-associated vasculitis, anti-glomerular basement membrane disease, and thrombotic microangiopathy [75]. The rapid removal of these large molecules by TPE reduces morbidity and mortality.

The first study to describe the use of plasmapheresis for the treatment of severely complicated wasp sting, including AKI, was published in 1988 [76]. Noteworthily, PLA1 (34 kDa), PLA2 (15 kDa), antigen 5 (23 kDa), hyaluronidase (38 kDa), and myoglobin (16.7 kDa), as well as inflammatory mediators such as IL-6 (21 kDa), IL-10 (16 kDa), IL-17 (19 kDa), IFN-α (19 kDa), and IFN-β (23 kDa), which are known to contribute to wasp venom-induced AKI, are all large molecular weight molecules. Theoretically, TPE could postpone or even prevent AKI in these cases by removing these pathological molecules from the plasma that are present in the venom. To prevent ongoing damage to organs, early removal of these molecules is undoubtedly a better strategy. However, small- or medium-sized toxins cannot be cleared efficiently by TPE alone. Patients with wasp venom-induced AKI have increased serum urea nitrogen and creatinine levels, generally accompanied by hyperkalemia and volume overload. Because the plasma separation membranes required by TPE are compatible with continuous renal replacement therapy (CRRT) systems, TPE coupled with CRRT is commonly used as a combination therapeutic approach. More recently, growing evidence has provided insights into the rational use of this combined treatment strategy. Zhang et al. [77] reported that patients treated with TPE + CRRT can obtain more rapid and better recovery of kidney function than those treated with intermittent hemodialysis; therefore, they suggested the use of TPE for patients with severe rhabdomyolysis who have high levels of CK and myoglobin [77]. In addition, our previous findings indicated that TPE + CRRT can reduce the levels of inflammation mediators (IL-8 and tumor necrosis factor-α) and markers of rhabdomyolysis (CK, LDH, and myoglobin) [78]. Particularly, the combination of TPE + CRRT was more effective at reducing the levels of inflammatory mediators and rhabdomyolysis markers, as well as the Sequential Organ Failure Assessment score, than hemoperfusion + CRRT [78]. More recently, Liu et al. [79] found that TPE treatment significantly decreases the mortality rate of wasp sting patients compared with those not treated with TPE; however, CRRT and hemoperfusion did not significantly reduce mortality [79]. According to these results, the TPE + CRRT combination appears to be a more suitable therapy option for wasp venom-induced AKI [11,79]. Nevertheless, doctors and nurses should be aware of the adverse events of TPE, such as hemorrhage, particularly in the case of wasp sting patients with abnormal coagulation. Moreover, high-quality clinical studies are warranted to explore more effective treatments.

### 5.2. Potential therapeutic drugs

#### 5.2.1. Cilastatin

Cilastatin is a renal dehydropeptidase I inhibitor previously used in clinical anti-infective therapy in combination with the broad-spectrum antibiotic imipenem. Combination with cilastatin aimed to prevent imipenem hydrolysis by renal dehydropeptidase I, thereby improving imipenem stability while reducing its nephrotoxicity and maintaining its antibacterial activity [80,81]. In addition, previous *in vitro* and *in vivo* studies showed that cilastatin has renal protective effects against nephrotoxic drugs, including cisplatin, gentamicin, vancomycin, and calcineurin inhibitors [82–84]. Cilastatin also has a protective effect against non-drug kidney damage, such as ischemia-reperfusion-induced AKI [85]. Further studies have found that megalin is the target of cilastatin in the kidney, which is expressed in the apical membrane of renal proximal tubular epithelial cells and plays a key role in the tubular reabsorption of various toxic substances mediating glomerular filtration [86]. Cilastatin has been proven to have nephroprotective effects in an animal model of glycerol-mediated rhabdomyolytic AKI by blocking the reabsorption of myoglobin by megalin [87]. Our recent *in vivo* study on wasp venom-induced AKI showed that cilastatin can significantly reduce the nephrotoxicity of wasp venom by blocking the reabsorption of myoglobin in renal tubular cells by megalin, reducing the concentration of myoglobin in the blood, and increasing the clearance of myoglobin in the urine [41]. Moreover, the nephroprotective effects of cilastatin in wasp venom-induced AKI are associated with improved oxidative stress, inhibition of the inflammatory response, attenuation of apoptosis, inhibition of complement hyperactivation, and reduction of nephrotoxic myoglobin accumulation. Considering that cilastatin has been widely used in clinical practice for several decades and its safety has been confirmed, cilastatin is expected to become a potential therapeutic drug for wasp sting-induced AKI.

#### 5.2.2. Varespladib

Varespladib, a nonspecific PLA2 inhibitor, was originally developed to treat acute coronary syndrome [88]. Both wasp and snake venom contain PLA2, which was identified as an ideal toxin-specific target for snake bites [89]. Notably, Lewin et al. [90] found that varespladib inhibits the PLA2 activity of various snake venoms [90]. Further studies have indicated that myotoxicity, nephrotoxicity, neurotoxicity, and mortality caused by different snake venoms can be prevented by varespladib [91]. Hence, varespladib has been used in clinical studies on snakebite treatment [89]. More recently, *in vitro* and *in vivo* studies demonstrated that varespladib also significantly inhibits PLA2 activity in wasp venom, ultimately preventing glycerophospholipid hydrolysis of muscle cells, red blood cells, and renal tubular epithelial cells [42]. Moreover, varespladib exhibits nephroprotective effects in a wasp sting-induced AKI model by ameliorating hemolysis, rhabdomyolysis, renal function, and pathological injury [42]. Based on these findings, varespladib holds promise as a potential therapeutic agent for the treatment of wasp venom-induced AKI.

## 6. Conclusions and prospects

AKI is a serious complication of multiple wasp stings, and is associated with a high mortality rate. Currently, its pathogenesis remains unclear and can only be said to progress gradually. Existing clinical reports on AKI induced by wasp venom are usually observational and retrospective, with a limited number of patients. The available *in vivo* models provide an effective tool for exploring the underlying mechanism and potential therapeutic agents for wasp venom-induced AKI. Based on a recent study, myoglobin released by rhabdomyolysis and myoglobin-induced inflammation response may play a central role in wasp venom-induced AKI, but the specific mechanism needs to be further investigated. To prevent further damage to the body by wasp venom, myoglobin, and inflammatory factors, patients with severe wasp stings should be hospitalized as soon as possible and closely monitored. Currently, the combined use of CRRT and TPE appears to be the preferred approach for treating AKI resulting from wasp stings. However, robust clinical evidence, such as data from multicenter randomized controlled trials, is still necessary to support this treatment strategy. Adsorption therapy, such as CytoSorb (which target 5–60 kDa proteins), may theoretically be used to remove the myoglobin, inflammatory mediators, and other components triggered or transported within the wasp venom, but the high financial burden associated to this approach limits its used to treat wasp venom-induced AKI. To date, no report has explored the use of CytoSorb in patients stung by wasps [92]. Regarding the use of cilastatin and varespladib in the treatment of wasp venom-induced AKI, existing studies indicate their potential as nephroprotective agents; nonetheless, further research is needed to determine their clinical applicability.

## Data Availability

Data sharing is not applicable to this article as no new data were created or analyzed in this study.

## Abbreviations

AIN: acute interstitial nephritis
AKI: acute kidney injury
ATN: acute tubular necrosis
CK: creatine kinase
CRRT: continuous renal replacement therapy
LDH: lactate dehydrogenase
IFN: interferon
IL: interleukin
MCP: monocyte chemotactic protein
mtDNA: mitochondrial DNA
PLA: phospholipase A
STING: stimulator of interferon genes
TPE: therapeutic plasma exchange.
